# A Simple Mechanism Causing Wealth Concentration

**DOI:** 10.3390/e22101148

**Published:** 2020-10-13

**Authors:** Michał Cieśla, Małgorzata Snarska

**Affiliations:** 1 M. Smoluchowski Institute of Physics, Jagiellonian University, ojasiewicza 11, 30-348 Kraków, Poland; michal.ciesla@uj.edu.pl; 2 Department of Financial Markets, Cracow University of Economics, Rakowicka 27, 31-510 Kraków, Poland

**Keywords:** wealth condensation, agent-based computational economics, bargaining, gain function

## Abstract

We study mechanisms leading to wealth condensation. As a natural starting point, our model adopts a neoclassical point of view, i.e., we completely ignore work, production, and productive relations, and focus only on bilateral link between two randomly selected agents. We propose a simple matching process with deterministic trading rules and random selection of trading agents. Furthermore, we also neglect the internal characteristic of traded goods and analyse only the relative wealth changes of each agent. This is often the case in financial markets, where a traded good is money itself in various forms and various maturities. We assume that agents trade according to the rules of utility and decision theories. Agents possess incomplete knowledge about market conditions, but the market is in equilibrium. We show that these relatively frugal assumptions naturally lead to a wealth condensation. Moreover, we discuss the role of wealth redistribution in such a model.

## 1. Introduction

Study of wealth distribution among the population has been labelled as one of the key problems in modern economic theory and is often described by a power-law function known as Pareto distribution [1]. In this sense, research related to wealth distribution and wealth inequalities is two-fold. The well-studied macro-perspective focuses on the issue of poverty arising from wealth inequalities, its social and economic consequences, where it is typical that a small fraction of the population owns most of the total wealth. This approach stems from macroeconomic theory and general equilibrium (c.f. [2]) like the infinite-lived dynasty model [3] and overlapping generations model [4]. Other concepts refer to asymmetric knowledge [5], a different number of connections or opportunities to exchange or increase wealth [6] or only to luck or different competencies [7]. Most of these models usually rely on representative agent paradigm, while completely ignoring immanent aspects of human nature and psychological biases or even microstructural characteristics of trade mechanism [8]. On the other hand, micro-foundations of wealth concentration arise from bilateral trade or exchange of goods among two economic agents, where wealth typically is highly related to the individual investment decision process. This observation led to several mathematical models attempting to explain this phenomenon, i.e., so-called kinetic wealth exchange models that are based on microeconomic interactions between economic actors who exchange wealth between them over the trade cycle [9]. These include models introduced by Angle [10], Bennati [11], Chakraborti and Chakrabarti [12], Dragulescu and Yakovenko [13] and recently also the approaches by Vallejos et al. [14] and Lim and Min [15], which share some common features with our approach.

### 1.1. Some Stylised Facts Related to Empirical Wealth Distribution

A well-known fact about wealth distribution in developed-economy states is that wealth is highly concentrated and very unequally distributed. Data sets gathered over 30 years by Census Survey of Consumer Finances confirm via, e.g., observation of historical trends that a degree of wealth concentration in the United States is high, i.e., almost one-third of total wealth is kept by only 1% of households, while the top 5% of the population holds more than one half of total wealth. At the other edge, there is a large fraction of the community, who has pretty little wealth or no wealth at all. These results are quite persistent over time, and substantial changes in net wealth are subject to a boom-bust cycle of financial and economic crises [16]. Little work has, however, been done in the area of understanding mechanisms leading to wealth concentration during economic upturns and equalisation effects during recessions. We contribute to this field by introducing new kinetic wealth exchange model with simplified assumptions, that can reproduce stylised facts observed in the empirical distribution of wealth in the crisis and post-crisis times. As a starting point, we use a real-life example of wealth distributions in the U.S. in the years 2010 (crisis) and 2018 (post-crisis). We believe that our model can shed light into better recognition of patterns leading to changes in wealth inequalities during the real business cycle.

**Figure 1:**
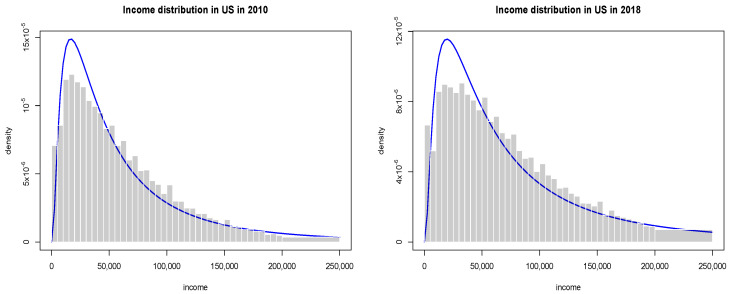
The empirical distribution of wealth in the U.S. based on Census Data. The blue line represents one of fitted generalised gamma distribution of the second kind.

Although in economics income is typically defined as the amount of money an economic agent or household receives on regular basis and wealth is related to the length of time that a family could maintain their current lifestyle without receiving compensation for performing additional work, we treat these two categories as a whole. The primary source of our data on wealth in the U.S. for our empirical examples is the U.S. Bureau of Census and Bureau of Labour Statistics Current Population Survey for Household Income from the years 2010 and 2018. The survey has been conducted monthly for over 50 years, with over 54,000 households selected based on an area of residence to represent the nation as a whole, individual states, and other specified areas. Each family is interviewed once a month for 4 consecutive months one year, and again for the corresponding period a year later. These data are however available only in a binned or aggregated form, so the only available data include the number of households in each bin, mean income, standard error and income limits assigned to each bin. To estimate income or wealth probability density function, we use the entropy-based divergence method and seek a probability density function, that is as close to the uniform distribution as the data sample will permit [17,18] (see the Appendix A). Results of estimation are presented in Figure 1. In Table 1, we have also gathered Gini coefficients and information criteria, for various distributions from the Creesse and Read (C.R) family [19].

Results presented in Table 1 and Table 2 confirm that empirical distributions of wealth exhibit a Pareto power-law tail

(1) $$f\left(x\right)∼\frac{1}{x^{1+\alpha }}\;1<\alpha <2,$$

and the actual shape of distribution at intermediate values of wealth is well fitted by a generalised gamma distribution of the second kind. So they can be reproduced by a simple kinetic wealth exchange model with either homogeneous or heterogeneous agents [9]. Furthermore, as seen in Figure 1, the post-crisis inequalities are larger than the crisis ones.

### 1.2. The General Structure of Kinetic Exchange Models

In this section, we will briefly review the basic features of kinetic wealth exchange models following [9,14,15]. As usual, the economy is assumed to consist of *N* agents with wealth $\left\{a_{k}\geq 0\right\}$ (k=1,2,…,N). At each cycle, an agent *i* exchanges a quantity Δa of wealth with another agent *j*. Both agents are chosen randomly. The total wealth $X=\sum_{i}a_{i}$ and the average wealth 〈a〉=X/N are constant. After the wealth exchange, $a_{i}$ and $a_{j}$ are updated according to the rule:

(2) $$\begin{array}{ccc} a_{i}^{\prime } & = & a_{i}-\Delta a\;,\\ a_{j}^{\prime } & = & a_{j}+\Delta a\;, \end{array}$$

under the condition ($a_{i}^{\prime },a_{j}^{\prime }\geq 0$). The signs have been chosen without the loss of generality and the function $\Delta a=\Delta a(a_{i},a_{j})$ is responsible for the dynamics of the underlying wealth concentration mechanism. Furthermore, agents can be parametrised by a maximum fraction of wealth ω∈(0,1] that enters each cycle or exchange process, which determine the time scale of the relaxation process and the mean value 〈a〉 at equilibrium [9]. If the value of ω is identical for all agents, then models belong to a homogeneous class that can reproduce the shape of the gamma wealth distribution. For ω<1, models converge toward a stable state with a wealth distribution with non-zero median, and for diversified agents, a power-law tail behaviour can be recovered. If $\omega_{k}$ is different for every agent, then models are called heterogeneous.

In the Angle model [10], changes of wealth between agents are determined by

(3) $$\begin{array}{c} \Delta a=r\;\omega \;[\eta_{ij}\;a_{i}-\left(1-\eta_{ij}\right)\;a_{j}]\;, \end{array}$$

where random variable r∈(0,1) is distributed uniformly or with a certain probability distribution g(r), and $\eta_{ij}$ is a random dichotomous variable responsible for the direction of the changes. The value $\eta_{ij}=1$ produces a wealth transfer $\left|\Delta a\right|=r\;\omega \;a_{i}$ from agent *i* to *j*, while the value $\eta_{ij}=0$ corresponds to a wealth transfer $\left|\Delta a\right|=r\;\omega \;a_{j}$ from *j* to *i*.

Another model is a One-Parameter Inequality Process model [20]

(4) $$\begin{array}{c} \Delta a=-\eta_{ij}\omega a_{i}+\left(1-\eta_{ij}\right)\omega a_{j}\;, \end{array}$$

where $\eta_{ij}=0$ or $\eta_{ij}=1$ are chosen randomly in each cycle. In these models, wealth distribution is best described by a gamma distribution.

Bennati [11] proposed a model, where agent exchange constant amount of wealth $\Delta a_{0}$ and transaction between agent *i* and *j* take place if and only if $a_{i}^{\prime },a_{j}^{\prime }\geq 0$, which leads to an exponential distribution of wealth.

Chakraborti and Chakrabarti [12] introduced a model, where new wealth $a_{i}^{\prime }$ ($a_{j}^{\prime }$) is expressed as a sum of the saved fraction $\lambda a_{i}^{\prime }$ ($\lambda a_{j}^{\prime }$) of the initial wealth and a random fraction *r* ($\bar{r}$) of the total remaining wealth, obtained summing the respective contributions of agents *i* and *j*.

(5) $$\begin{array}{c} \Delta a=\omega \left(\bar{r}\;a_{i}-r\;a_{j}\right)=\left(1-\lambda \right)\left(\bar{r}\;a_{i}-r\;a_{j}\right)\;. \end{array}$$

Dragulescu and Yakovenko introduced another model [13] with dynamics described as:

(6) $$\begin{array}{c} \Delta a=\bar{r}\;a_{i}-r\;a_{j}\;, \end{array}$$

leading to an exponential model for wealth distribution.

Lim and Min [15] consider various kinetic exchange models in search for solidarity effects consider multiple variants of the model introducing heterogeneous savings parameter $\lambda_{k}$ and wealth dependent trading rules

(7) $$\begin{array}{c} \Delta a=r\min(a_{i},a_{j}) \end{array}$$

with *r* being random variable uniformly distributed over [0,1] and updated every transaction.

The common factor of all these models is the wealth conservation: $a_{i}+a_{j}=a_{i}^{\prime }+a_{j}^{\prime }$, which means that while one agent gains money from a transaction, the other one has to lose some wealth. Therefore, without any preference of richer agents over the poorer ones and due to random character of interactions between agents, these kinetic exchange models can be characterised by a stationary wealth distributions, which exhibit exponential tails. After reaching this distribution wealth, inequalities do not increase anymore. It should be noted that there are also kinetics exchange models that exhibit power-law wealth distribution as a stationary state [21,22]. These models often assume some preferences of richer individuals; for example, individuals’ wealth is repeatedly multiplied by a random factor, different for each individual.

Another type of agent model is one with a growing economy, where wealth is continuously added to the system and divided among the agents. One such model has been used recently by Vallejos et al. to study the growth of wealth inequalities in the U.S. [14]. There the assumption is, however, that individuals with greater wealth get significantly more of this added wealth than poorer agents. Namely, this wealth is divided into several equal parts, and each part is given to the agent *i* with probability proportional to $a_{i}^{\beta }$. In this setup, Authors studied how the initial Pareto like wealth distribution changes depending on β. For 0<β<1, the power of wealthier individuals is diminished much more than the power of poorer individuals and wealth inequalities lowered over time. For β=1, the model gives all individuals a proportional amount of power, and thus initial wealth distribution does not change. For β>1, the model provides a disproportionate amount of power to the wealthier individuals in the market, and the wealth inequalities grew.

To sum up, the discussed agent models reaches a stationary state when wealth inequalities do not increase any more or exhibit constant grow of wealth concentration under the assumption that richer agents are disproportionately better treated than the poorer ones. In this study, we propose a model of trading agents where inequalities grow incessantly, but the gain of each agent is at average proportional to its wealth, which corresponds to the case of β=1 in Vallejos et al. model [14], where inequalities do not change.

## 2. Model

In our model, an agent is a participant of a market game. An agent can be a company, an institution as well as a single person. Agents can trade with themselves using their assets. It is worth noting that for our study “assets” are not only goods or money that an agent has, but also widely understood services he can make or even its skills that allows him to be more effective in a market. Agents can interact with themselves, which affects their assets. In general, such interaction covers all possible activities like, for example, buying or selling products, services, financial market instruments, etc., as well as making money at work or employee hiring. From our perspective, all activities mentioned above are indistinguishable, so we will call each of them using the same word—a trade. As mentioned, a simple example of trade is the buying of a product in a shop. Another example is hiring an employee by a company. Trade is also when one agent exchanges his knowledge with another one.

In a further study, we have considered two assumptions:

(i) agents are equal in the sense that each of them has the same access to the market and the same knowledge about it.
(ii) agent trade only when it is profitable from their perspective.

These assumptions are quite general. The first one reflects capabilities given by a modern technology where at least virtual access to goods, financial markets and stock-exchanges is common. Therefore the number of transaction that agent can make is limited only by the number of his assets. The second assumption corresponds to decision and utility theories, which tell us that action will be undertaken by the individual only when it causes maximisation of the individual utility [23,24]. It is worth noting that typically utility and assets are not the same quantities. They are not even measured in the same units. However, we think that they can be somehow compared to the money, which measures assets, spent to increase utility. Therefore there is a relation between these two concepts, and in further considerations, we will treat the utility as an asset.

The society consists of $N=10^{5}$ individuals (agents). Each of them possesses some assets. Let $a_{i}$ denotes a share of *i*-th agent assets in the whole population wealth. Thus

(8) $$\sum_{i=1}^{N}a_{i}=1.$$

Agents interact with themselves, which affects their wealth. As stated before a trade is a win-win situation, i.e., both trading agents gain a profit from it. Because a market is in equilibrium and all agents have comparable information about it, their profits from a single trade should also be comparable. Here, we assume that profits from trade are equal for both agents and are given by a deterministic gain function g(i,j)=g(j,i), where *i* and *j* are the trading agents. Thus, the trade changes the trading agent’s assets as follows:

(9) $$\begin{array}{c} a_{i}\to a_{i}+g\left(i,j\right)\\ a_{j}\to a_{j}+g\left(j,i\right) \end{array}$$

The above, fully deterministic, rules reflect the second condition made in the Introduction section. The first condition about equal access to the market is fulfilled by a specific matching process between two bargaining agents. They are selected according to their wealth. Thus the probability that *i*-th agent will be chosen for trading is equal to $a_{i}$. It reflects the fact that wealthier agents have more opportunities to trade, but on the other hand, it does not exclude poorer ones from the market.

Trades are grouped into cycles. A single cycle contains N/2 trades. The protocol used for choosing agents involving in trade is as follows

1. The first agent *i* is chosen randomly with the probability equal to its wealth $a_{i}$.
2. The second agent *j* is chosen randomly with the probability equal to its wealth $a_{j}$.
3. If i=j or agent *i* has traded with agent *j* in this cycle, go to point 1. Otherwise, make the trade.

Each pair of agents can trade at most once during a single cycle. This prevents a situation that all the trades are only between the richest individuals, and thus, increase the chance of gaining profits by poorer agents. (Without this assumption, two richest agents may perform all the trades. However even with this restriction, it may occur that $\sqrt{N}$ number of agents will concentrate all the wealth and they will trade with themselves only, but it is not possible to limit further the number of trading agents.) After each cycle, individuals portfolios of assets are normalised to fulfil condition (8)

(10) $$a_{i}\to \frac{a_{i}+\sum_{j}g\left(i,j\right)}{1+\sum_{i,j}g\left(i,j\right)},$$

where $\sum_{j}$ sums all profits made by the *i*-th agent, and $\sum_{i,j}$ sums all profits from all transactions within a cycle. To fully specify the model, a particular gain function g(i,j) is needed. The simplest, symmetrical functions of two variables is a constant function: g(i,j)=r/N, i.e., each agent receives a lump sum of money during a single trade. Note that the number of trades depends on agents wealth—richer individuals trades more because they split their wealth into a larger number of transaction. Thus, each transaction in the model involves the same amount of assets, and therefore, a constant payoff is justified. Although it might seem that such a mechanism is similar to preferential treatment of some agents as in other models e.g., [25], we will show in the following section that it is not in the case of this model. The payoff *r* was typically set to 0.1. Note that *r* is equal to the global income from all trades within a cycle when the global wealth is equal to 1. Therefore the specific value of *r* determines the speed of wealth distribution changes between cycles.

## 3. Results and Discussion

### 3.1. Wealth Condensation

**Figure 2:**
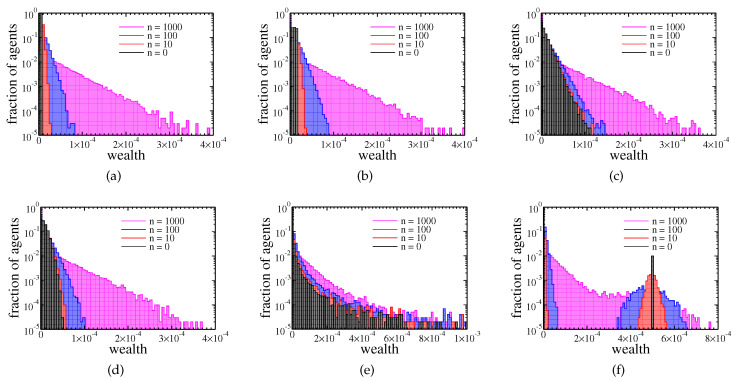
Histograms of agents assets after 0, 10, 100, and 1000 cycles. Different plots correspond to the different initial distribution of wealth among the agents: $a_{i}$ is (**a**) equal to 1/N, (**b**) uniformly distributed in the interval [0, 2/N), (**c**) exponentially distributed, (**d**) normally distributed, (**e**) Cauchy distributed, (**f**) 1% of richest agents possess 100 times more than the rest 99% of agents.

The model was tested numerically. The evolution of wealth distribution is presented in Figure 2 (See the software used for simulations and data analysis in Supplementary).

The plots differ in initial wealth distribution among agents. Here, we used the following distributions.

(a) delta distribution—all agents started with the same amount of money;
(b) uniform distribution—the initial wealth of each agent was uniformly distributed on the interval [0,2/N);
(c) exponential distribution—the initial wealth was drawn according to the exponential distribution of the unit mean and variance;
(d) Gaussian distribution—the initial wealth of each agent was an absolute value of a number drawn according to the normal distribution of the zero mean value and unit variance;
(e) Cauchy distribution—the initial wealth of each agent was an absolute value of a number drawn according to the following probability distribution function
  (11) $$p\left(x\right)=\frac{1}{\pi (x^{2}+1)};$$
(f) 1% of richest agents possessed 100 times more money than the remaining 99% of poorer agents.

After choosing initial wealth distribution, the assets of each agent were normalised to fulfil condition (8).

**Figure 3:**
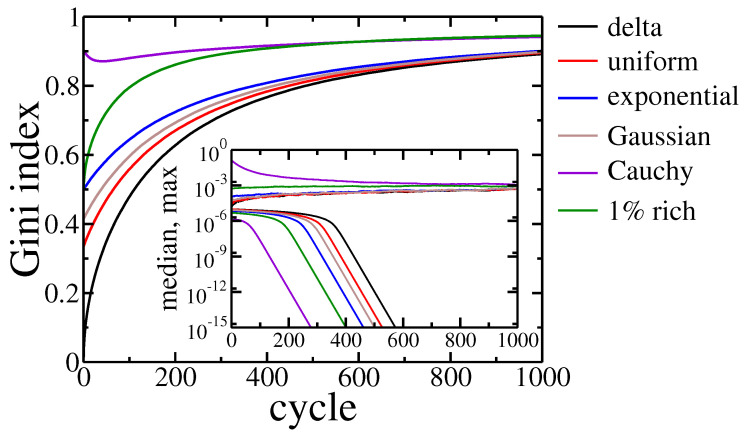
Gini index evolution for all studied agents initial wealth distributions. Inset shows the evolution of the maximal and median wealth in the population of agents. Black line corresponds to equal initial wealth of all agents, red—the uniform distribution of wealth on the interval [0,2/N), blue—the exponential wealth distribution, brown—Gaussian distribution, violet—Cauchy distribution, green—1% of richest agents have 100 bigger assets than the rest 99% of the population.

In most of these cases, the final wealth distribution (after 1000 cycles) was almost the same. Only in the last case we did not obtain purely exponential distribution after 1000 cycles, but here, the wealth distribution also should converge to an exponential distribution with a growing number of cycles. These results suggest that the wealth condensation was a feature of the model and not of the specific initial wealth distribution among agents. The effect of wealth condensation was confirmed by analysis of the Gini coefficient:

(12) $$G=\frac{2\sum_{i=1}^{N}ia_{i}}{N\sum_{i=1}^{N}a_{i}}-\frac{N+1}{N},$$

as well as the wealth of the richest and the middle agent (see Figure 3).

For most of the studied cases, the Gini index grew monotonically with the evolution of the system. The only exception was when initial wealth was drawn according to Cauchy distribution, which is an example of power-law, long-tail distribution. Here, the Gini index started from a relatively high value, as the condensation was an intrinsic property of power-laws. However, after the initial decrease corresponding to recombination to exponential distribution preferred by the model, it started to grow again. It is worth noting that the richest agent in this scenario lost most of its initial assets, but a rapid decrease of the median asset in the population (see Figure 3 inset) shows that wealth condensation occurred anyway. It should be stressed that the final state of these simulations was not stable in either case. The wealth inequalities seemed to grow endlessly. After 1000 cycles the Gini coefficient was above 0.89, and the richest agent owned approximately $5.3\times 10^{-4}$ of the total wealth and the median wealth several orders of magnitude smaller.

**Figure 4:**
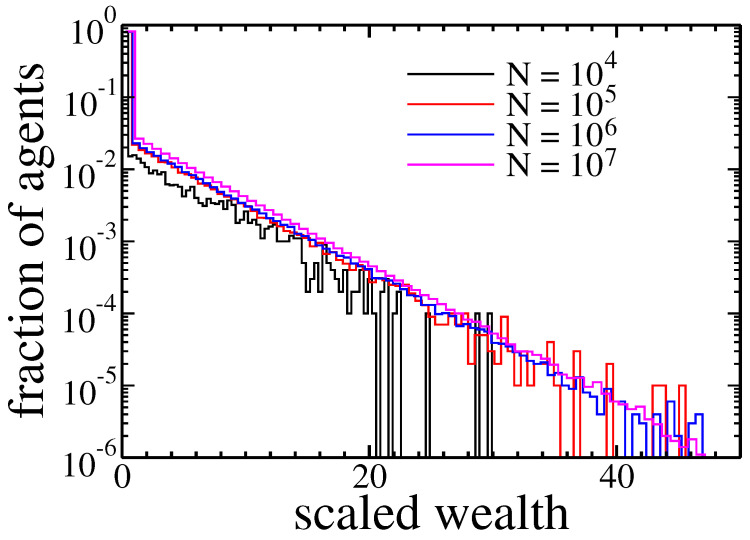
Histograms of agents assets after 1000 cycles for different size of the population. Because the total wealth changes with population size, we rescaled agents wealth by multiplying it by the number of agents *N*.

Next, we check if the population size affected condensation. It was done by studying systems consisting of $N=10^{4}$ up to $10^{7}$ agents—see Figure 4.

After 1000 cycles all populations exhibited a similar, exponential distribution of wealth.

At last we checked if the appearance of wealth condensation does not depend on model details. Therefore, instead the g(i,j)=r/N we studied numerically other examples of payoff functions. In particular we performed independent analysis of the model using the following different utility functions, namely:

(i) linear preferences utility function: $g\left(i,j\right)=r\frac{a_{i}+a_{j}}{2}$—gains from individual transaction depend on assets of both sides of trade process.
(ii) Cobb–Douglas utility function: $g\left(i,j\right)=r\sqrt{a_{i}a_{j}}$—similar as in the above case but gains were much lower when agents assets differed significantly.
(iii) Koopmans and Leontieff utility function: $g\left(i,j\right)=r\min\left(a_{i},a_{j}\right)$—gains are determined by a poorer trader.

**Figure 5:**
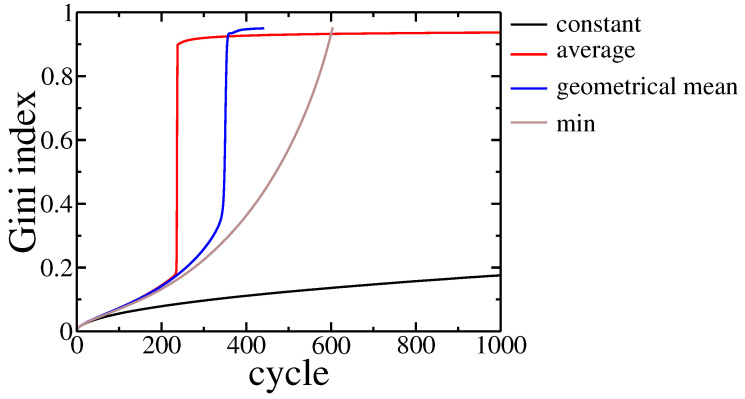
Gini index evolution for different payoff function used in the model. The black line corresponds to constant payoffs while the other ones correspond to payoffs depending on trading agents assets: red—payoff proportional to average assets of trading agents, blue—payoff proportional to geometrical mean of trading agents assets, brown—payoff proportional to poorer agent asset. The parameter r=0.01 and population size $N=10^{5}$.

Results are presented in Figure 5.

In all studied cases, we observed wealth condensation. It occurred even faster than for the constant gain, as the above functions give additional profits from transactions between richer agents, which are more probable within the studied model. Moreover, for Cobb–Douglas and Koopmans–Leontieff functions, a small group of agents took all the assets, and thus, further evolution, according to model rules, became impossible. The model rules assume that in a single cycle there were N/2 trades between different pairs of agents. However, these agents were chosen with probability given by their assets, and thus there was practically no opportunity to draw randomly one of the poor agents as their wealth was negligibly small. Even if such trade occurred the assets of the poorer did not change significantly due to properties of these utility functions.

Because the wealth condensation occurred for all studied cases of the initial wealth distribution, we further focused on the case where all agents had an equal lump of money initially, and the population size was $10^{5}$. To get some insight into obtained results, we analysed a simpler model, where an agent could trade with himself, and there was no restriction that each pair could trade at most once during a cycle. Note that these rules were more generous for a richer agent than the ones used in numerical simulations. We checked numerically that these restrictions had no qualitative influence on the phenomenon of wealth concentration as well as the type of wealth distribution. In such a case, each agent had *N* independent opportunities to trade, and each option was used with probability equal to the agent’s wealth. Thus, the number *k* of *i*-th agent transactions during the cycle is given by the binomial distribution:

(13) $$p_{i}\left(k\right)=\left(\begin{array}{c} N\\ k \end{array}\right)a_{i}^{k}{\left(1-a_{i}\right)}^{N-k}.$$

The gain of *i*-th agent after the cycle is kr/N, and the total gain of all agents is *r*. Thus, after normalisation (see Equation (10)) the wealth of the *i*-th agent will be

(14) $$a_{i}\to a_{i}+\Delta a_{i},$$

where

(15) $$\Delta a_{i}\left(k\right)=\frac{r}{1+r}\left(\frac{k}{N}-a_{i}\right).$$

The mean value of such binomial distribution is $\langle k\rangle =Na_{i}$, and the variance $var\left(k\right)=Na_{i}\left(1-a_{i}\right)$. Because $\Delta a_{i}$ is a linear function of *k*, its mean value equals to

(16) $$\langle \Delta a_{i}\rangle =\frac{r}{1+r}\left(\frac{Na_{i}}{N}-a_{i}\right)\equiv 0.$$

This result depended neither on a particular value of $a_{i}$ or distribution of wealth among agents. It means that in this model, share of wealth of each agent, at average, remained constant. It corresponds to the case β=1 in the Vallejos et al. model [14], where the initial wealth distribution was stable. Remembering that the presented here model had additional restrictions limiting the number of transactions mainly for the richest individuals, the observed concentration of wealth is therefore highly unexpected. To give some explanation of this phenomenon, we studied the variance of $\Delta a_{i}$, which is equal to

(17) $$var\left(\Delta a_{i}\right)=\langle {\left(\Delta a_{i}\right)}^{2}\rangle ={\left(\frac{r}{1+r}\right)}^{2}\frac{a_{i}\left(1-a_{i}\right)}{N}.$$

The variance is a square function of the agent’s wealth $a_{i}$ and has a maximum for $a_{i}=0.5$. In our model, the initial value of $a_{i}$ was typically much smaller than 0.5 due to a large number of agents, so in that case, it was safe to assume that for poorer individuals the wealth would change slower than for richer ones. In other words, if someone became poor, it would be tough for him to regain his wealth. However, if a relatively small group of agents accumulated wealth that made the probability significantly higher (for the whole group) than 0.5, that the wealth of this group, as a whole, would be conserved due to decreasing variance for $a_{i}>0.5$. This may be the mechanism which stabilises inequalities caused initially by random fluctuations.

### 3.2. Income and Wealth Tax Influence on the Model

**Figure 6:**
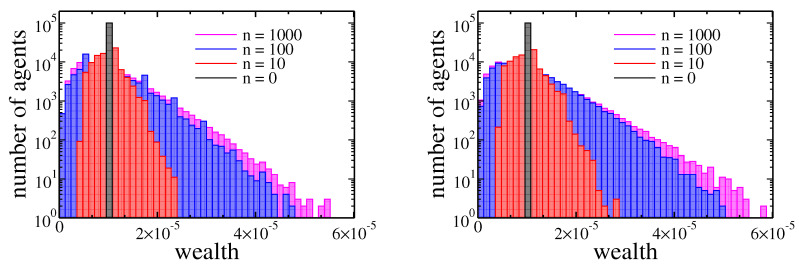
Histograms of agents assets after 0, 10, 100, and 1000 cycles. In both plots, the wealth of agents was initially equal ($a_{i}=10^{-5}$). The left plot corresponds to income tax ($t_{I}=0.1$ and $t_{W}=0$), and the right one corresponds to wealth tax ($t_{I}=0$ and $t_{W}=0.01$).

Other important aspects of wealth concentration are the redistribution effect and optimal taxation problem [26,27]. To check how taxes influence the results of our exchange game model we analysed two different approaches to trade taxation. One was based on a linear income tax, i.e., where tax was collected within a single trade cycle, and the other on wealth tax, and affected total holdings of each agent collected over multiple trading cycles. In both cases, the tax was collected from all the agents and then was distributed equally among them. Thus the Equation (10) becomes:

(18) $$a_{i}\to \frac{\left(1-t_{W}\right)a_{i}+\left(1-t_{I}\right)\sum_{j}\frac{r}{N}+\frac{t_{W}+t_{I}r}{N}}{1+r},$$

where $\Delta a_{i}=\sum_{j}\frac{r}{N}$ is an income of *i*-th agent during one cycle, and $t_{I}$ and $t_{W}$ are income and wealth tax rates, respectively. Note that in the above relation we took into account that the global income was equal to *r* while the total wealth was normalised to 1. Due to the latest opinions that only wealth tax can lower wealth inequalities [27,28,29], we are particularly interested in comparing these taxes within our model. Therefore we studied two different situations—pure income tax with tax rate set at 10% ($t_{I}=0.1$ and $t_{W}=0$), and pure wealth tax with rate set at 1% ($t_{I}=0$ and $t_{W}=0.01$). For r=0.1 such choice of rates causes redistribution of 1% of the global wealth per cycle in both cases. In general, to get the same global income from the wealth tax and the income tax, the ratio of their rates should be *r*. The comparison of both cases is presented in Figure 6.

**Figure 7:**
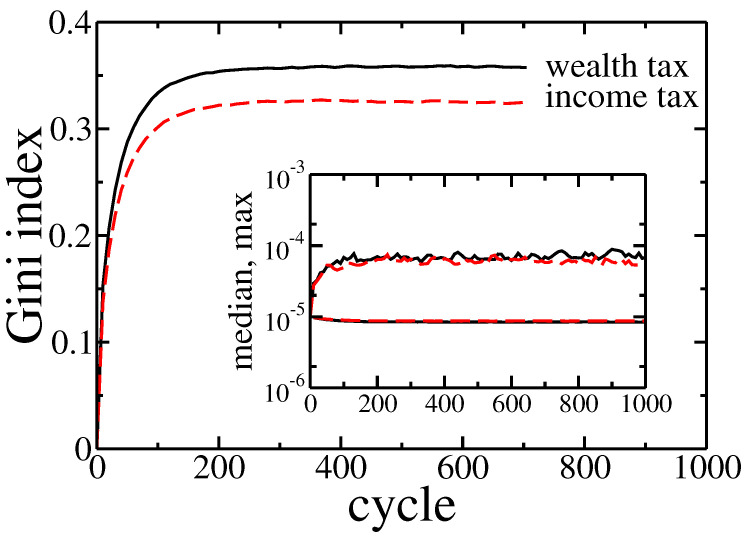
Gini index evolution for pure wealth (solid black line) and income (dashed red line) tax applied to the model. Initially, the wealth was distributed equally among agents ($a_{i}=10^{-5}$). Inset shows the evolution of the maximal and median wealth in the population of agents.

In fact there was no significant difference between these two kinds of taxation. The detailed analysis of the Gini coefficient (see Figure 7) suggests that the wealth tax followed to slightly larger inequalities than the income tax. It is in contradiction with above-mentioned, well-established opinions [27,28,29]. On the other hand, this effect is quite easy to explain. Existence of wealth condensation meant that the relation of income to the wealth was effectively higher for richer individuals. Thus, the linear income tax hit the rich more than the wealth tax. However, in a real economy, it is easier to hide or reduce declared income than wealth. Therefore, in general, the wealth tax can be more effective as easier to enforce.

**Figure 8:**
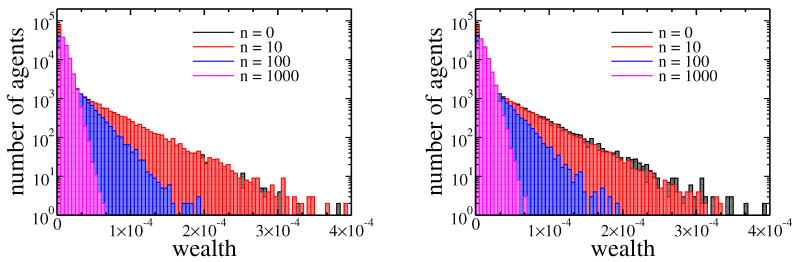
Histograms of agents assets after 0, 10, 100, and 1000 cycles. In both plots the initial wealth of agents was taken from the final state of simulation (1000 cycles) presented in a Figure 2. The left plot corresponds to income tax ($t_{I}=0.1$ and $t_{W}=0$), and the right one corresponds to wealth tax ($t_{I}=0$ and $t_{W}=0.01$).

Until now we have shown that within our model there were no significant differences between income and wealth based redistribution when initially wealth was equally distributed among agents. However, in a real society, we never have equally distributed goods. Therefore, it is particularly interesting how these two taxes affects the evolution of wealth in the case of high inequalities, such as, for example, one presented in Figure 2 obtained after 1000 cycles. The evolution of wealth in this case is presented in Figure 8.

**Figure 9:**
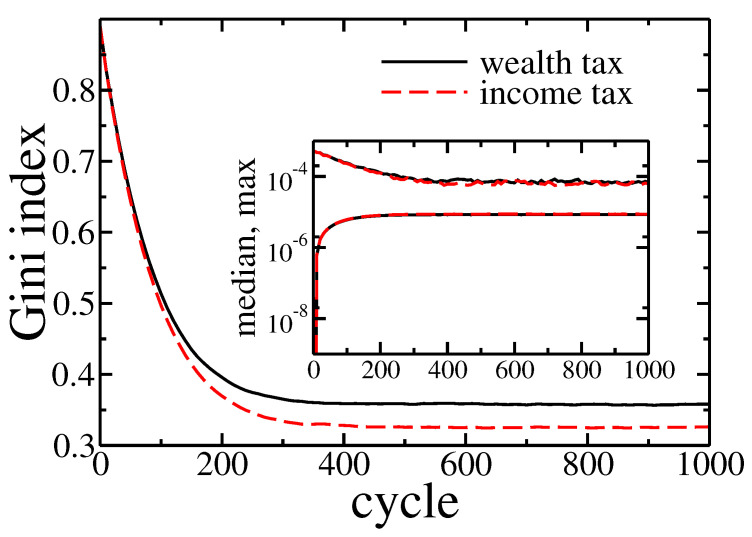
Gini index evolution for pure wealth (solid black line) and income (dashed red line) tax applied to the model. The initial wealth of agents was taken from the final state of simulation (1000 cycles) presented in Figure 2a. Inset shows the evolution of the maximal and median wealth in the population of agents.

Note that, in contrast to previous cases the wealth distribution shrank with time (growing number of cycles). Again however, there was no significant difference between income and wealth based redistribution. All these observations were confirmed by Gini coefficient evolution presented in Figure 9.

In the beginning, wealth inequalities and the Gini coefficient was high, but wealth redistribution quickly tamed them. As previously, the income tax was slightly more effective. What is also important, the final state reached after 1000 cycles was similar to one with obtained for equal wealth distribution at the beginning of simulations—note the horizontal scale difference between Figure 2 and Figure 8. It allows trusting, that this final state was universal and did not depend on initial conditions. In both cases, the median wealth increased by several orders of magnitude. For example, for income tax based redistribution, the median raised from $2.1\times 10^{-33}$ to $8.7\times 10^{-6}$ at the cost of richest agent wealth, which decreased from $5.9\times 10^{-4}$ down to $6.2\times 10^{-5}$, and the 90% of total wealth was owned by 74% of richest agents. What is even more spectacular, only 10 cycles were needed to raise the median to $6.1\times 10^{-7}$ at a cost of the richest agents wealth decreasing down to $4.7\times 10^{-4}$.

**Figure 10:**
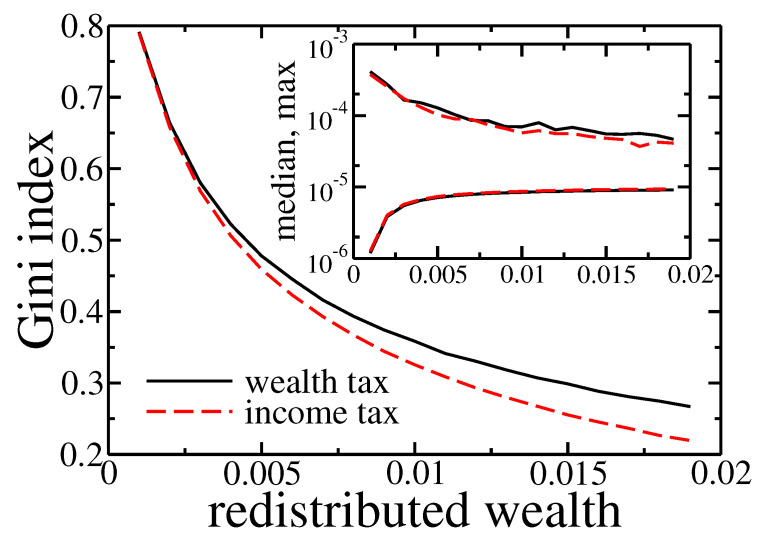
Gini index after 1000 cycles for wealth (solid black line) and income (dashed red line) tax based redistribution. The initial wealth of agents was taken from the final state of simulation (1000 cycles) presented in Figure 2. Inset shows final maximal and median wealth in the population of agents.

To ultimately prove that within presented model redistribution based on the income and wealth taxes gives similar effects we analysed the dependence of Gini coefficient after 1000 cycles on redistributed amount of wealth, for high wealth inequalities at the beginning of simulations. Results are presented in Figure 10.

As expected, inequalities dropped down with growing redistribution. Moreover, the final value of Gini coefficient, the richest agent wealth and median of wealth in the society depended on the amount of redistributed wealth but they almost did not depend on a type of applied tax.

## 4. Conclusions

We proposed a simple mechanism owing utility theory, in which an individual with more assets has more opportunities to interact with others, but, at average, the gain from these interactions is proportional to individual’s wealth. Despite this, using agent-based modelling, we showed that this mechanism causes wealth condensation independently on details of the studied model as well as its initial condition. The observation has been also supported by analytic arguments. In contrast to kinetic exchange models e.g., [10,11,12,13,15], here, the wealth inequalities grow for a large range of initial conditions and this growth is not limited by a specific distribution with exponential tails. In contrast to other models with growing economy [14], no disproportionately better treatment of wealthier agents is required to fuel the growth of wealth inequalities.

It suggests that the phenomenon of wealth condensation can be much more fundamental than expected, as it may appear even in the absence of any form of disproportionately preferential treatment of some groups of individuals.

We also studied the influence of wealth redistribution based on income and wealth taxes within the model. It occurs that while the level of inequalities depends on the amount of redistributed wealth, it almost does not depend on the type of applied tax.

## Figures and Tables

**Table 1:** Information criteria for fitted distributions.

Distribution	2010			2018		
	Gini	Aic	Bic	Gini	Aic	Bic
Generalised Beta of the Second Kind	0.4504	825,368.9827	825,407.7613	0.4656	914,590.8355	914,629.8928
Generalised Gamma	0.4485	825,598.8432	825,627.9271	0.4526	915,341.0957	915,370.3886
Beta of the Second Kind	0.4545	825,501.8130	825,530.8969	0.4636	915,233.3365	915,262.6295
Dagum		1,258,915.0143	1,258,944.0982	0.4693	914,642.1348	914,671.4277
Singmad	0.4531	827,239.3961	827,268.4800	0.4600	914,833.1633	914,862.4562
Lognormal	0.5013	832,408.2444	832,427.6337	0.5206	924,094.3485	924,113.8772
Weibull	0.4432	827,065.1604	827,084.5496	0.4462	916,179.0877	916,198.6163
Gamma	0.4409	826,112.8345	826,132.2238	0.4467	915,559.4152	915,578.9439
Doubly lognormal		1,375,275.6949	1,375,295.0841		920,090.4281	920,109.9568
Pareto	0.5047	832,191.8408	832,211.2301	0.5061	920,845.1144	920,864.6431

**Table 2:** Estimated distribution parameters: location μ, scale σ, skewness ν and kurtosis τ.

Distribution	2010				2018			
	μ	σ	ν	τ	μ	σ	ν	τ
Generalised Beta of the Second Kind	108,564.1708	1.7786	0.7034	2.0083	113,253.3847	2.1917	0.5323	1.2229
Generalised Gamma	60,663.6500	0.9001	0.7612		81,899.1321	0.9057	0.8421	
Beta of the Second Kind	283,044.3149	1.5608	7.5992		372,137.5280	1.4877	7.2873	
Dagum	1,012,451,669.9591	0.9721	0.1021		105,486.1708	2.4436	0.4689	
Singmad	1,012,451,669.9591	1.1348	53,697.3940		190,335.2758	1.3413	3.3841	
Lognormal	10.6958	0.9900			10.9507	1.0373		
Weibull	69,527.7650	1.1699			89,843.3170	1.1589		
Gamma	6,5806.3882	0.8652			85,510.9515	0.8789		
Doubly lognormal	1,012,451,669.9591	0.1514			60,235.5085	1.7064		
Pareto		1,549,526.7678	24.2996					

## Supplementary Materials

The following are available online at https://www.mdpi.com/1099-4300/22/10/1148/s1. The software used for simulations and data analysis is attached to the article.

## Appendix A

To find estimation and inference measures that will enable linking the model to a family of possible likelihood functions related to income data, we use a single parameter family of entropic function-power divergence measures given by [17]:

(A1) $$I\left(p,q,\gamma \right)=\frac{1}{\gamma (\gamma +1)}\sum_{i=1}^{n}p_{i}\left[{\left(\frac{p_{i}}{q_{i}}\right)}^{\gamma }-1\right]\;,$$

with γ being a parameter indexing Creesse and Read (C.R.)-entropy family of divergence measures-distributions, $p_{i}$ are probabilities that need to be estimated, and $q_{i}$ are reference probabilities from a uniform distribution.

Estimation of income distribution from a sample of real data is then equivalent to the solution of the optimisation problem [18]:

(A2) $$\hat{p}=\arg\min_{p}\left[I\left(p,q,\gamma \right)\sum_{j=1}^{n}p_{j}d_{j}\right];\;\sum_{j=1}^{n}p_{j}=1;\;p_{j}\geq 0\;,$$

where $d_{j}$ is a discrete random income variable, representing mean income with *j*-th bin. In the limit criterion γ→0, the problem further converges to:

(A3) $$\max_{p}\left[-\sum_{j=1}^{K}p_{j}\log p_{j}\sum_{j=1}^{K}p_{j}d_{j}\right]\;,$$

which can be solved by via Lagrange multiplier $\hat{\lambda }$ leading to

(A4) $${\hat{p}}_{j}=\frac{\exp(-d_{j}\hat{\lambda })}{\sum_{j=1}^{K}\exp\left(-d_{j}\hat{\lambda }\right)}\;.$$

This procedure is part of a statistical generalised additive model for location, scale and shape. In principle, a parametric distribution, which might be heavy-tailed and positively skewed, is assumed for target variable and distribution can vary according to explanatory variables using smooth functions. This distribution is characterised by location μ, scale σ and remaining parameters are shape parameters, i.e., skewness ν and kurtosis τ [19]. To select a proper parametrisation *M* for wealth distribution, we apply Akaike (aic) and Bayes (bic) information criteria.

(A5) bic(M)=klog(n)−2logLaic(M)=2k−2logL

with *k* equal to the number of model parameters, *n* being a sample size, and *L* being the maximised likelihood function and look for a distribution model *M* with minimal values of either bic or aic.
